# CAGNet: A Network Combining Multiscale Feature Aggregation and Attention Mechanisms for Intelligent Facial Expression Recognition in Human-Robot Interaction

**DOI:** 10.3390/s25123653

**Published:** 2025-06-11

**Authors:** Dengpan Zhang, Wenwen Ma, Zhihao Shen, Qingping Ma

**Affiliations:** School of Mechanical and Power Engineering, Henan Polytechnic University, Jiaozuo 454000, China; 212205010032@home.hpu.edu.cn (W.M.); shenzhihao1001@hpu.edu.cn (Z.S.); maqingping@home.hpu.edu.cn (Q.M.)

**Keywords:** Facial Expression Recognition, Multiscale Feature Aggregation, Convolutional Block Attention Module, Global Average Pooling

## Abstract

The development of Facial Expression Recognition (FER) technology has significantly enhanced the naturalness and intuitiveness of human-robot interaction. In the field of service robots, particularly in applications such as production assistance, caregiving, and daily service communication, efficient FER capabilities are crucial. However, existing Convolutional Neural Network (CNN) models still have limitations in terms of feature representation and recognition accuracy for facial expressions. To address these challenges, we propose CAGNet, a novel network that combines multiscale feature aggregation and attention mechanisms. CAGNet employs a deep learning-based hierarchical convolutional architecture, enhancing the extraction of features at multiple scales through stacked convolutional layers. The network integrates the Convolutional Block Attention Module (CBAM) and Global Average Pooling (GAP) modules to optimize the capture of both local and global features. Additionally, Batch Normalization (BN) layers and Dropout techniques are incorporated to improve model stability and generalization. CAGNet was evaluated on two standard datasets, FER2013 and CK+, and the experiment results demonstrate that the network achieves accuracies of 71.52% and 97.97%, respectively, in FER. These results not only validate the effectiveness and superiority of our approach but also provide a new technical solution for FER. Furthermore, CAGNet offers robust support for the intelligent upgrade of service robots.

## 1. Introduction

With the rapid advancement of artificial intelligence, service robots are becoming increasingly prevalent across various domains, including companionship, daily life assistance, communication, and many others, thus becoming an integral part of human daily life [1]. For example, in healthcare settings, service robots can adapt their interaction strategies by recognizing changes in human facial expressions, providing better support to patients [2]. Similarly, in educational environments, these robots can optimize teaching strategies based on students’ facial feedback, ultimately enhancing the learning experience [3]. Consequently, to more effectively understand and respond to human emotional needs, it is essential for service robots to possess efficient Facial Expression Recognition (FER) capabilities, which enables robots to accurately capture and interpret the emotional cues conveyed through facial expressions to offer the user more personalized and empathetic services [4,5].

FER methods generally consist of three key stages: face detection, feature extraction, and classification. Among these, feature extraction is the most critical step. Accurately extracting features that genuinely reflect expression-related information is essential to improve the accuracy of FER [6]. In recent years, the extensive application of deep learning technology has greatly promoted the progress of this field. In particular, the advantages of Convolutional Neural Networks (CNNs) in automatic feature extraction have significantly improved the recognition performance [7].

Although deep learning has made remarkable progress in the field of FER, the recognition accuracy of many existing models on standard datasets still has room for improvement, especially in the recognition of complex expression changes and subtle emotional differences. Some current mainstream network structures often struggle to fully integrate local details and global information during the feature extraction process, resulting in insufficiently comprehensive expression feature representation. Therefore, how to design a network structure that can effectively enhance feature expression and improve recognition accuracy has become an important direction in current research.

To address the above issues, this paper proposes a new FER network, CAGNet. It enhances the model’s ability to recognize subtle expression changes through multi-scale feature fusion and attention mechanisms, and introduce attention mechanisms to strengthen the expressive power of key facial regions. Experimental results show that the network achieves recognition accuracy superior to existing methods on two public datasets, providing technical support for service robots to achieve more precise emotional perception.

The structure of this paper is organized as follows. Section 2 reviews relevant research works in the field of FER. Section 3 introduces the methodology and the proposed network architecture. Section 4 presents the experimental results. Section 5 discusses the experimental outcomes and their applications in robotics. Finally, Section 6 concludes the paper and outlines future research directions.

## 2. State-of-the-Art

Traditional FER algorithms typically rely on manually designed feature extractors including Local Binary Patterns (LBPs) [8], Histogram of Oriented Gradients (HOGs) [9], Gabor wavelet transforms [10], Haar-like features [11], and others. Researchers [12] introduced a face detection method based on Haar-like features combined with an AdaBoost cascade classifier, demonstrating relatively good detection performance for frontal face images under well-lit conditions. However, due to its dependence on manually designed features, the method’s ability to effectively capture facial expressions from multiple viewpoints requires further enhancement. Zhang et al. [13] proposed a method for multi-scale and multi-directional filtering of images. At each scale, the maximum pixel values across eight directions at each coordinate are fused to form a new feature map. Subsequently, classification was performed using histogram-based block statistics, which integrated richer features while reducing computational complexity. Traditional facial recognition research methods, although capable of achieving good results in recognition and classification, were highly dependent on handcrafted features. This not only consumed a significant amount of time and effort but also made the system’s performance heavily reliant on the choice of features. The reliance on handcrafted features led to poor system stability, as these features were susceptible to interference from external factors.

To overcome these limitations, deep learning techniques have been widely adopted in the field of FER in recent years. Deep learning simplifies the feature extraction process and removes the need for manual features design by automating the process of learning high-level feature representations from raw data. In particular, CNN is capable of automatically capturing both local and global features in images [14]. Through multiple layers of convolution and pooling operations, CNN progressively extracts more abstract feature representations. Examples of deep neural network models that have been widely used include AlexNet [15], VGGNet [16], GoogLeNet [17], ResNet [18], and DenseNet [19]. Furthermore, the introduction of Batch Normalization (BN) layers, dropout layers [20,21], and regularization techniques into the CNN architecture can further enhance the model’s performance and generalization capabilities, making it better suited for complex application scenarios. Zhang et al. [22] proposed a novel FER algorithm based on an improved residual neural network. By designing a residual neural network and introducing the Mish activation function and Inception module to extract deep features, significant performance improvements have been achieved on multiple datasets. Xie et al. [23] introduced a deep residual network model that combined attention mechanisms with deformable convolutions. The model embedded position-aware attention mechanisms within residual blocks and incorporated deformable convolutions at both input and output layers, enhancing the capability to extract features from different positions in the image. Liang et al. [24] proposed a lightweight FER method that combined an improved CNN with channel weighting. The method used a neural network architecture that combined standard and depthwise separable convolutions, and employed a Global Average Pooling (GAP) layer as the output layer. By introducing the Squeeze-and-Excitation (SE) module for channel weighting, the accuracy of the network model was enhanced. Zhang et al. [25] enhanced the traditional VGG network by adding BN layers to accelerate convergence and improve generalization. The model was validated on four datasets and achieved excellent results. Zhao et al. [26] presented a lightweight FER model based on knowledge distillation. This model greatly increased the accuracy of the lightweight student model by optimizing the distillation loss function. Guo et al. [27] proposed an efficient self-healing network model, which performed linear gather classification for label rectification after fusing features by using a multi-scale attention method and capturing and weighting facial expression regions. Yang et al. [28] proposed a robust driver emotion recognition method based on feature separation. This method can effectively overcome the influence of individual differences and light changes through partial feature exchange and multi-loss constraint mechanisms, and has performed excellently on both self-built and public datasets. Chudasama et al. [29] presented a multimodal fusion network for dialog emotion recognition, which integrates text, audio, and visual features through a multi-head attention fusion layer, introduces a novel feature extractor and adaptive margin triplet loss to optimize feature learning, and designs a weighted facial expression model to combine scene and facial information. On multimodal datasets, the weighted average F1 score of this model outperforms the existing methods.

Although deep learning has made remarkable progress in the field of FER, such as improving feature extraction and classification effects through models like CNN and Recurrent Neural Networks (RNNs), these technologies still face problems such as complex models and low accuracy. Meanwhile, simple cascade structures may ignore the complex interactions between high-level and low-level features, making it difficult to capture subtle differences in expressions. Therefore, exploring more efficient network structures and optimization strategies to overcome these limitations has become an important direction in current research [30].

This paper introduces CAGNet, a novel network that combines multiscale feature aggregation and attention mechanisms to enhance FER. CAGNet employs a hierarchical convolutional architecture to effectively extract multi-scale features from input images. By integrating Convolutional Block Attention Module (CBAM) and GAP modules, it optimizes the capture of both local and global features, improving sensitivity to subtle expression differences. BN layers and Dropout techniques are also incorporated to enhance model stability and generalization, preventing overfitting.On the FER Challenge 2013 (FER2013) and Extended Cohn-Kanade dataset (CK+) standard datasets, CAGNet achieves recognition accuracies of 71.52% and 97.97%, respectively, outperforming traditional network models and other related methods.Furthermore, the FER algorithm has been deployed on service robots, enabling them to perceive users’ emotional states. Combined with the MediaPipe Pose [31] algorithm for real-time human posture detection, this system enables intelligent control of robotic movement.

## 3. Methodology

### 3.1. CBAM Attention Mechanism

**Figure 1:**
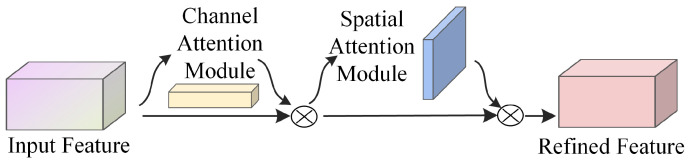
CBAM network structure.

When observing objects, humans tend to selectively focus on more important information while ignoring less relevant parts, a phenomenon known as attention. In deep learning, the introduction of attention mechanisms allows models to concentrate on key information and ignore less important details, thereby emphasizing and retaining the features that are crucial for the task. This paper primarily adopts a hybrid attention mechanism in computer vision, where the CBAM is one of the most representative [32]. CBAM integrates channel attention and spatial attention mechanisms in a sequential manner, allowing for a comprehensive analysis of the input feature maps across both channel and spatial dimensions. This ensures that feature regions receive thorough attention. The network architecture is depicted in Figure 1. where “Input Feature” and “Refined Feature” represent the input feature map and the refined feature map, respectively.

**Figure 2:**
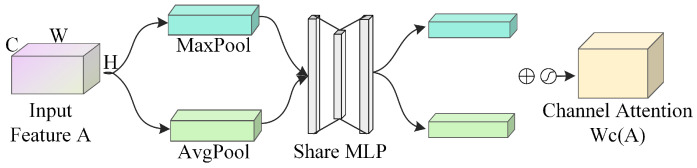
Channel attention mechanism network structure.

The channel attention mechanism operates by assigning varying weights to individual channels, with higher weights signifying greater importance for a given channel. As shown in the structure of the channel attention mechanism in Figure 2, an input feature map *A* of dimensions H×W×C is compressed along the spatial dimensions using both Average Pooling (AvgPool) and Maximum Pooling (MaxPool), resulting in two spatial descriptor features, $A_{c\_avg}$ and $A_{c\_max}$, each of size 1×1×C. Following dimensionality reduction and expansion, these compressed feature descriptors are input into a single hidden layer Multilayer Perceptron (MLP) to extract weight vectors that represent the relative relevance of each channel. The two feature vectors processed by the MLP are summed up, and the resulting vector is passed through a Sigmoid activation function (denoted as σ for the Sigmoid operation) to produce the channel attention weight coefficients $W_{c}(A)\;$. The channel-attended feature map $A^{\prime }$ is then created by multiplying these channel attention weight coefficients element-wise by the original input feature map.

(1) $$A_{c\_\mathrm{avg}}=\mathrm{Avgpool}(A)\;$$

(2) $$A_{c\_\max}=\mathrm{Maxpool}(A)\;$$

(3) $$W_{c}(A)\;=\sigma (\mathrm{MLP}(A_{c\_\mathrm{avg}})+\mathrm{MLP}(A_{c\_\max}))$$

(4) $$A^{\prime }=W_{c}(A)\;⨂A$$

**Figure 3:**
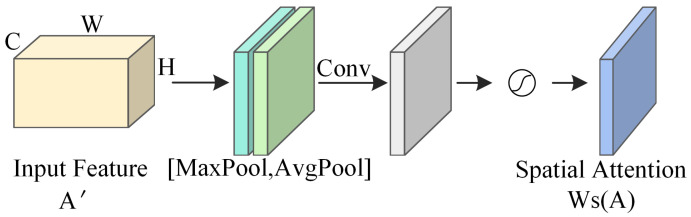
Network architecture of the spatial attention mechanism.

In the FER task, employing a spatial attention mechanism can effectively retain the key information in facial expression images that are affected by rotation, distortion, and scale changes, thereby mitigating the negative impacts brought about by these transformations. As depicted in Figure 3, the network structure of the spatial attention mechanism processes the input feature map $A^{\prime }$ through Avgpool and Maxpool operations along the channel dimension, generating two efficient feature descriptors, $A_{s\_avg}$ and $A_{s\_max}$. These descriptors are then concatenated horizontally and subjected to a convolution operation using a 7×7 kernel *f*7×7 to capture spatial contextual information. The output after convolution is passed through a Sigmoid activation function (denoted as σ for the Sigmoid operation), producing the spatial attention weight coefficients $W_{s}(A)\;$. Lastly, the input feature map is multiplied by the spatial attention weight coefficients to produce the spatial attention feature map $A^{\prime \prime }$.

(5) $$A_{s\_\mathrm{avg}}=\mathrm{Avgpool}(A^{\prime })$$

(6) $$A_{s\_\max}=\mathrm{Maxpool}(A^{\prime })$$

(7) $$W_{S}(A)\;=\sigma (f^{7\times 7}([A_{s\_\mathrm{avg}};A_{s\_\max}]))$$

(8) $$A^{\prime \prime }=W_{S}(A)\;⨂A^{\prime }$$

### 3.2. Global Average Pooling

**Figure 4:**
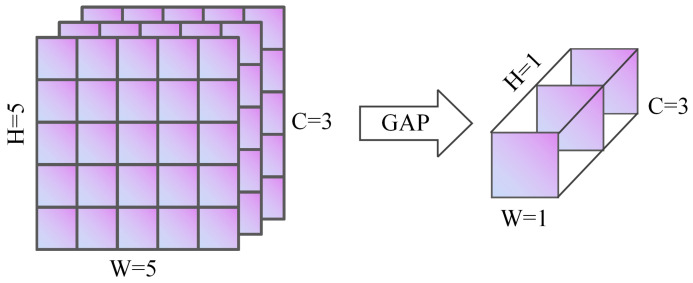
Schematic Diagram of global average pooling.

In CNNs, GAP is a technique that effectively reduces model parameters, avoids overfitting, and improves generalization ability [33]. Different from traditional Fully Connected Layers (FC), GAP generates a fixed-length vector by calculating the pixel average value of each feature map channel. It not only retains key feature information, but also makes the model more lightweight and easier to train. Its parameters are adaptively determined by the spatial dimensions of input feature maps, eliminating the need for manual setting of *H* and *W*, which shows good structural simplicity and generalization performance [34]. As shown in Figure 4, when a feature map of size H×W×C is input into the network, GAP computes the average of all H×W pixel values for each channel, resulting in a 1×1×C vector. Tasks like image classification, object identification, and semantic segmentation are made easier by the fact that this vector can be used directly for classification tasks or routed through an FC or Softmax layer to obtain the final classification output.

(9) $${\mathrm{GAP}(X)}_{c}=\frac{1}{H\times W}\sum_{i=1}^{H}\sum_{j=1}^{W}X_{i,j,c}$$

### 3.3. The Proposed Network

**Figure 5:**
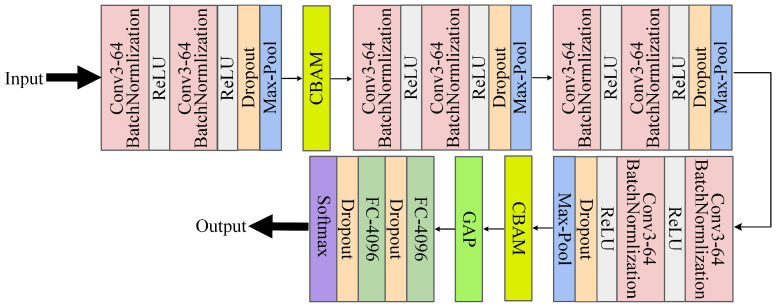
Architecture of CAGNet.

Every convolutional module in CAGNet uses a multi-layered structure, with a stack of two convolutional layers coming first, followed right away by a BN layer and a ReLU non-linear activation function. This architecture improves the model’s capacity for learning while guaranteeing that the data stays stable throughout transmission. L2 regularization, which lessens the size of the model’s weights and avoids overfitting, is integrated into both the convolutional and FC to enhance the model’s capacity for generalization. The data undergoes pooling operations after the two convolutional layers, batch normalization, and activation, which helps reduce computational complexity while retaining essential features. The CBAM attention mechanism is introduced after the first and fourth convolutional modules. This mechanism assigns weights to feature maps along both the spatial and channel dimensions, enabling the model to focus on emotion classification while filtering out irrelevant background information or distracting factors. Subsequently, global average pooling is applied to the feature maps, followed by FC and Dropout layers to refine feature extraction and classification. The Dropout rate is set to 0.1 after the activation function and 0.5 after the FC. The extracted features are then passed through a Softmax layer, which converts the features into probability distributions for each emotion category. Through the Softmax transformation, the model produces a probability vector, where each element represents the confidence level for the corresponding emotion category. Accurate facial emotion identification and classification are achieved by using the index of the maximum value in the output vector to determine the emotion state conveyed by the input facial image. Figure 5 depicts the architecture of CAGNet.

In this architecture, multi-layer convolution and pooling are applied to process 48 × 48 grayscale input images. After each 3 × 3 convolutional layer and 2 × 2 pooling layer, the feature map size is halved sequentially. Following multiple convolution and pooling operations, the feature map dimensions are reduced from 48 × 48 to 24 × 24, then to 12 × 12, followed by 6 × 6, and finally to 3 × 3. GAP compresses the 3 × 3 feature map into a one-dimensional vector, which serves as input to the FC. The final emotion category probabilities are output through the Softmax layer. Table 1 provides a detailed breakdown of the entire processing flow from the input data to the final probability predictions of the emotion category in CAGNet.

## 4. Results

### 4.1. Experimental Data

To validate the effectiveness of the proposed CAGNet, which is a FER model based on multiscale feature aggregation and attention mechanisms, this study conducted experiments using the public datasets FER2013 [35] and CK+ [36].

**Figure 6:**
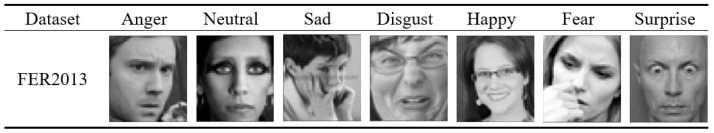
Examples from the FER2013 Dataset. Adapted the permission from ref. [35].

The images in the FER2013 dataset were obtained through Google and consist of facial images captured in natural settings, which were then uploaded to the internet. Consequently, the images may have issues such as occlusions, watermarks, variations in pose, and class imbalance. Using these datasets can demonstrate the robustness of the model. There are 35,887 photos of facial expressions in the FER2013 dataset, of which 28,709 are in the training set and 3589 are in each of the validation and test sets. As shown in Figure 6, it includes seven different types of facial expressions: Anger, Neutral, Sad, Disgust, Happy, Fear, and Surprise.

**Figure 7:**
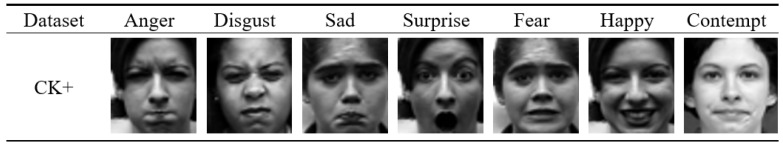
Examples from the CK+ Dataset. Adapted the permission from ref. [36].

The CK+ dataset is widely utilized and consists of images captured in a controlled laboratory setting, ensuring minimal interference from external factors. It contains 593 image sequences from 123 individuals, with 327 sequences labeled according to facial expressions. For this study, the last three frames from each sequence were selected, yielding a total of 981 expression images, which were subsequently resized to 48 × 48 pixels. The CK+ dataset includes seven expression labels: Anger, Disgust, Sad, Surprise, Fear, Happy, and Contempt, as shown in Figure 7.

Image preprocessing was employed on both datasets to improve the trained model’s capacity for generalization and reduce the possibility of overfitting brought on by insufficient data. The images from the FER2013 and CK+ datasets were normalized to 48 × 48 pixel grayscale images, and the labels were one-hot encoded. Data augmentation techniques, including random scaling, horizontal and vertical translations, random rotations, and horizontal flipping, were applied to the images. The specific data augmentation strategies and their parameter configurations are shown in Table 2.

### 4.2. Experimental Procedure

The experiments in this study were conducted on a 64-bit Windows 10 operating system, using the deep learning framework TensorFlow-GPU 2.6.2 and the Python 3.7.16 interpreter. The hardware environment consisted of an AMD Ryzen Threadripper PRO 3945WX 12-Core CPU, an NVIDIA RTX A6000 GPU, and 64GB of RAM.

A batch size of 128 was used for training on the FER2013 dataset, and the model was trained across 300 epochs. The Stochastic Gradient Descent (SGD) optimizer with momentum was used to update the network parameters, and a value of 0.9 for momentum was chosen. A basic learning rate scheduler with an initial learning rate of 0.01 was incorporated into the Reduce Learning Rate on Plateau (RLRP) approach. For five consecutive epochs, the learning rate was lowered by a factor of 0.75 if the validation accuracy did not increase. In order to stop training if the validation loss did not improve after ten consecutive epochs, an EarlyStopping callback was also added. A batch size of 32 was employed for training on the CK+ dataset, and the model was trained across 200 epochs. An 8:2 ratio was used to randomly divide the dataset into training and testing sets. Additionally, the SGD optimizer with momentum was used, keeping the initial learning rate at 0.01 and the momentum value at 0.9.

### 4.3. Experimental Analysis and Comparison

#### Experimental Evaluation

The confusion matrix is used to assess CAGNet’s capacity to recognize different kinds of facial expressions. This matrix displays the expected labels on the horizontal axis and the actual labels on the vertical axis, while other cells show misclassifications between distinct expressions, the value at the intersection of a predicted and a true label represents the accuracy for that specific label. Therefore, the values along the main diagonal of the confusion matrix correspond to the correct classification rates for each expression. To enhance visual clarity, the depth of the blue color is used to represent the level of accuracy, with darker shades indicating higher accuracy. In general, higher values along the diagonal suggest lower recognition errors, while higher values off the diagonal indicate greater misclassification and higher recognition errors by the model.

**Figure 8:**
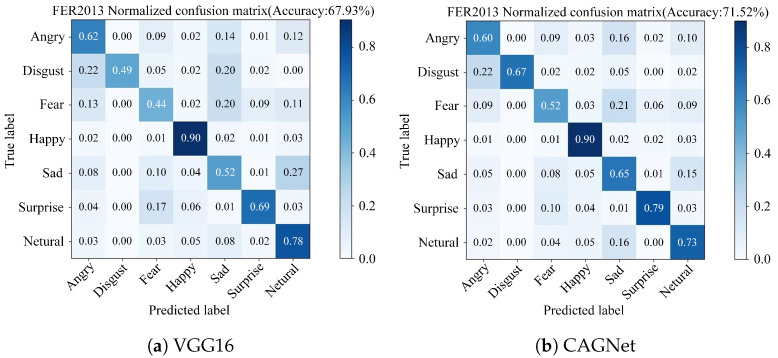
Confusion matrix for the FER2013 dataset. (**a**) VGG16. (**b**) CAGNet.

Based on the FER2013 dataset, this study compares the FER outcomes of CAGNet with those of the traditional VGG16 network, as illustrated in Figure 8. The results indicate that while the VGG16 network achieved an overall recognition accuracy of 67.93%, CAGNet reached an accuracy of 71.52%. Through this comparison, it is evident that the recognition accuracy for four categories of expressions Disgust, Fear, Sad, and Surprise have been notably improved. Specifically, the improvements for Disgust, Fear, Sad, and Surprise expressions are 18%, 8%, 13%, and 10%, respectively, while the accuracy for the Happy expression remains consistent. Furthermore, Angry expressions are prone to being misclassified as Sad, Netural or Fear. Disgust expressions tend to be misclassified as Angry. Fear expressions are likely to be incorrectly identified as Sad, Angry or Netural. Happy expressions have a tendency to be misrecognized as Neutral. Sad expressions can be misinterpreted as Neutral or Fear. Surprise expressions are often misclassified as Fear. Lastly, Neutral expressions are susceptible to being misidentified as Sad. In summary, CAGNet not only enhances the overall recognition accuracy but also reduces confusion errors for specific expression categories, namely Disgust, Fear, Sad, and Surprise, thereby demonstrating superior performance over the VGG16 network.

**Figure 9:**
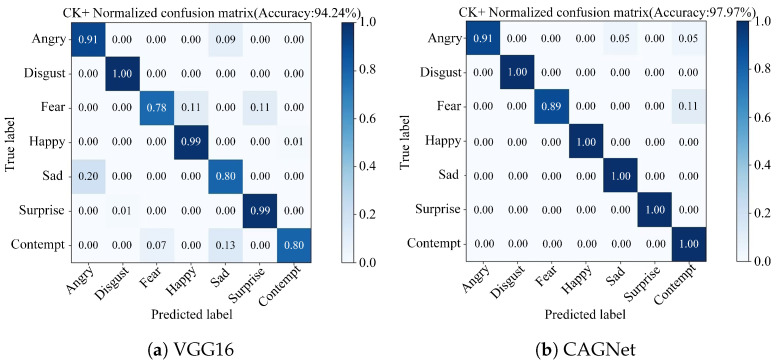
Confusion matrix for the CK+ dataset. (**a**) VGG16. (**b**) CAGNet.

Based on the CK+ dataset, the traditional VGG16 network achieved an overall recognition accuracy of 94.24%, whereas CAGNet reached an accuracy of 97.97%. As illustrated in Figure 9, the comparison reveals improvements in the recognition accuracy for Fear, Happy, Sad, Surprise, and Contempt expressions. Notably, the highest increases were observed for Sad and Contempt expressions, both of which improved by 20%. Meanwhile, the accuracy for Angry and Disgust expressions remained consistent. In terms of confusion across different expressions, Angry expressions are prone to being misclassified as Sad or Contempt, and Fear expressions are likely to be misclassified as either Contempt. Importantly, there are no confusion errors for Disgust, Fear, Happy, Sad, Surprise and Contempt expressions, indicating perfect classification within these categories. In summary, the proposed network not only enhances the overall recognition accuracy but also significantly reduces confusion errors for specific expression categories, namely Fear, Happy, Sad, Surprise and Contempt. This demonstrates the superior performance of CAGNet over the VGG16 network in terms of both accuracy and reduced misclassification rates across key expression categories.

## 5. Discussion

On the FER2013 dataset, the recognition accuracy for Happy expressions reaches as high as 90%, whereas the accuracy for Fear expressions is only 52%. This disparity is primarily attributed to the imbalance in the number of data samples within the test set and the lower quality of some samples. For instance, categories such as Happy and Neutral have significantly more samples compared to other categories. Additionally, the similarity between different expressions further complicates the model’s prediction performance. On the CK+ dataset, the highest recognition accuracies are achieved for Disgust, Happy, Sad, Surprise, and Contempt expressions, while the recognition performance for Angry and Fear expressions remains relatively poor. The subtle differences between these expressions make them difficult to distinguish, and potential imbalances or variations in sample quality within the dataset also impact the model’s performance.Overall, CAGNet consistently outperforms the traditional VGG16 network. This significant improvement can be attributed to several key enhancements: the introduction of an attention mechanism to focus on the most relevant features, the incorporation of BN layers to stabilize and accelerate the training process, and the addition of Dropout layers to reduce dependency on individual neurons and prevent overfitting, thereby enhancing the model’s generalization ability. Furthermore, GAP is employed to effectively reduce the number of model parameters, avoid overfitting, and strengthen the model’s generalization capability.

### 5.1. Comparison of Different Attention Mechanisms

Employing experiments on both the FER2013 and CK+ datasets, we performed a comparison study employing a consistent baseline network design to better examine the effects of various attention processes on model performance. We evaluated the performance of four attention mechanisms: Efficient Channel Attention Network (ECANet) [37], ClassAgnostic Segmentation Networks (CANet) [38], Squeeze-and-Excitation Networks (SENet) [39], and CBAM. The accuracy rates of these attention mechanisms on the two datasets are presented in Table 3. The experimental results show that ECANet achieved accuracy rates of 68.20% on FER2013 and 96.27% on CK+, CANet achieved accuracy rates of 67.98% on FER2013 and 95.93% on CK+, SENet achieved accuracy rates of 68.54% on FER2013 and 96.95% on CK+, and CBAM achieved accuracy rates of 71.52% on FER2013 and 97.97% on CK+. Although ECANet, CANet, and SENet also demonstrate good performance, they primarily enhance features in a single dimension (such as channel or spatial), and thus fail to fully exploit the interplay between different dimensions. In contrast, CBAM extracts complementary information among channels through global average pooling and max pooling operations in the channel dimension, and learns channel-wise weights using a shared network structure. In the spatial dimension, it compresses the channel information to generate a spatial attention map that highlights important regions in the image. By collaboratively enhancing feature responses in both the channel and spatial dimensions, CBAM can more comprehensively identify and strengthen semantic information in key facial regions, thereby improving the model’s ability to capture critical facial features. This leads to higher recognition accuracy, with outstanding performance on both the FER2013 and CK+ datasets.

### 5.2. Performance of the Proposed Network Model

To validate the effectiveness and superiority of CAGNet, a comparative study was conducted on the FER2013 and CK+ datasets with current mainstream FER methods. Li et al. [40] proposed an enhanced YOLOv8 algorithm to address challenges such as the difficulty of traditional convolutional operations in capturing subtle features and the effective fusion of multi-scale features. Kumar et al. [41] proposed a Cross-Connected Convolutional Neural Network (CC-CNN) for facial feature extraction. Lin et al. [42] proposed a FER method that combines an attention mechanism with the ShuffleNetV2 network. Boughida et al. [43] used Gabor filters and a genetic algorithm for expression recognition. Zhang et al. [22] proposed a novel FER algorithm based on an improved residual neural network. The comparison results are shown in Table 4. The model performance on the FER2013 dataset is generally lower than that on the CK+ dataset. This is mainly due to the fact that FER2013 contains low-resolution images, which may lack sufficient detail to distinguish subtle expression changes. Additionally, the FER2013 dataset suffers from class imbalance, with some categories having significantly more samples than others, which can lead to the model being biased towards the majority classes during training. In contrast, the CK+ dataset provides higher-resolution images and has a more balanced distribution across categories, resulting in generally better performance on these dataset.

### 5.3. Expression Recognition Application

**Figure 10:**
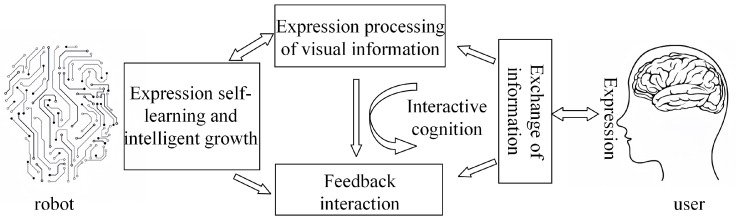
This relationship diagram of FER and robot collaboration.

The integration of FER technology with service robots can significantly enhance the quality of human-robot interaction. By recognizing users’ facial expressions, service robots can gain a better understanding of human emotional states, enabling them to provide more empathetic and personalized services. Users communicate emotional information through their facial expressions, which are captured by cameras or other sensors and processed by the system. The system then uses interactive cognitive mechanisms to interpret the user’s emotions and respond accordingly, fostering dynamic interaction. Throughout this process, the system continuously refines its accuracy and intelligence in FER through autonomous learning and adaptive growth, ultimately achieving a more natural, efficient, and personalized collaborative experience. The relationship between FER and robot collaboration is illustrated in Figure 10.

To enable service robots to understand and respond to users’ emotional states, this paper deploys FER algorithms onto a service robot platform and integrates them with a human pose perception module, thereby constructing an intelligent interactive system capable of both emotion perception and behavioral response. The service robot platform consists of an industrial control computer and a display terminal, serving as the core processing unit and human-machine interface. Environmental visual data captured by the camera is transmitted to the industrial computer for analysis and processing.

**Figure 11:**
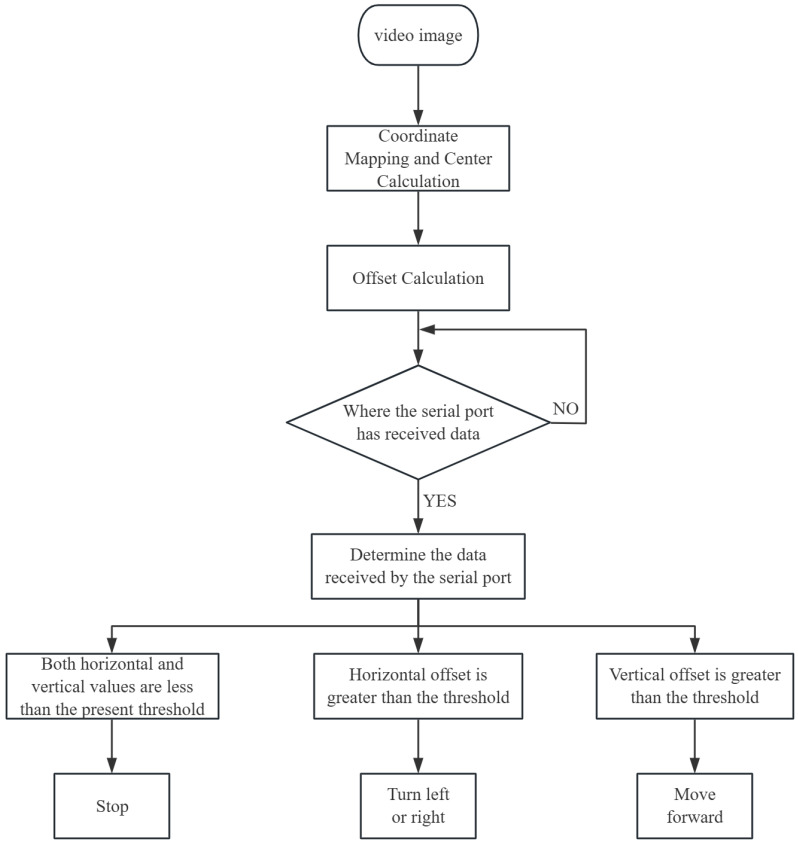
Robot tracking and control algorithm flowchart.

The system employs the MediaPipe Pose algorithm for real-time detection of human posture, extracting normalized coordinates of key body joints from the image and mapping them into the physical image space. Subsequently, a geometric averaging method is used to compute the central coordinate of the human posture, which helps determine the person’s spatial position within the image. When the horizontal offset between the human center point and the image center is less than a predefined threshold (50 pixels), the target is considered to be in the central region, and the robot receives a stop command. If the horizontal offset exceeds the threshold, left or right turning commands are generated based on the direction of the offset, enabling lateral following behavior. In the case of vertical offset beyond the threshold, a forward command is issued to guide the robot toward the user. All control instructions are transmitted to the robot’s lower-level control system via a serial communication protocol, enabling real-time parameter updates and action execution. This allows the robot to dynamically track the user’s position and perform autonomous motion control. A flowchart of the robot’s tracking control algorithm is shown in Figure 11.

**Figure 12:**
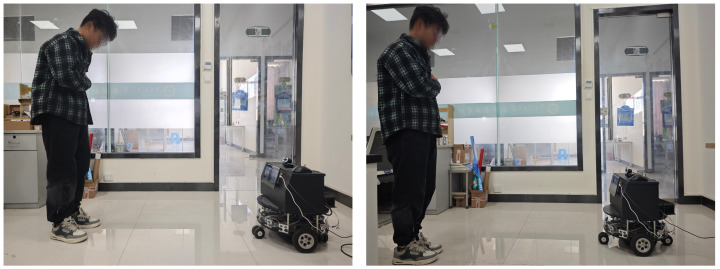
Tracking detection effect diagram of the service robot platform.

When the service robot detects a user’s facial expressions, it can perceive the user’s emotional state based on the recognition results. By integrating this capability with a human posture perception module for real-time localization and tracking, it further enhances the intelligence and humanization of human-robot interaction. Figure 12 showcases the tracking detection effect diagram of the service robot platform. The FER algorithm proposed in this paper cannot directly participate in the underlying force control of physical human-computer interaction, but it can serve as a high-level emotional auxiliary signal to enhance the system’s ability to understand users’ emotions. During the trajectory learning process based on Dynamic Movement Primitives, inputting emotional features as contextual information helps robots formulate differentiated response strategies according to different emotional states, thereby improving the interaction experience and safety in human-robot collaboration.The recognition performance of the current model may degrade under complex lighting conditions or partial facial occlusions. Meanwhile, its computational requirements also impose higher demands on resource-constrained edge devices.

## 6. Conclusions

In summary, this paper proposes CAGNet, a novel network that integrates visual techniques and combines multiscale feature aggregation with attention mechanisms, aiming to meet the demand for efficient expression recognition in service robots for production, companionship, and daily life service interactions. CAGNet employs a hierarchical convolutional structure, optimizing the capture of both local and global features through the integration of CBAM and GAP modules. Additionally, CAGNet incorporates BN layers and Dropout techniques to enhance its stability and generalization capabilities. Through rigorous evaluation on the FER2013 and CK+ standard datasets, the experimental results confirm the effectiveness of CAGNet, achieving recognition accuracies of 71.52% and 97.97%, respectively. This not only contributes a novel technical solution to the field of FER but also lays a solid technological foundation for the intelligent upgrade of service robots. In the future, more lightweight network architectures can be designed to adapt to embedded robotic platforms. Additionally, introducing multimodal information (such as speech and posture) can enhance the depth of emotional understanding and improve the model’s adaptability to complex scenarios with lighting changes and facial occlusions.

## Figures and Tables

**Table 1:** Detail structure of CAGNet.

Layer	Filter Size/Stride	Output SHAPE	Layer	Filter Size/Stride	Output Shape
Conv2d_1	3×3/1	48×48×64	max_pooling2d_3	3×3/2	6×6×256
Conv2d_2	3×3/1	48×48×64	Conv2d_7	3×3/1	6×6×512
max_pooling2d_1	3×3/2	24×24×64	Conv2d_8	3×3/1	6×6×512
CBAM_1	-	24×24×64	max_pooling2d_4	3×3/2	3×3×512
Conv2d_3	3×3/1	24×24×128	CBAM_2	-	3×3×512
Conv2d_4	3×3/1	24×24×128	GAP	-	512
max_pooling2d_2	3×3/2	12×12×128	FC1	4096	4096
Conv2d_5	3×3/1	12×12×256	FC2	4096	4096
Conv2d_6	3×3/1	12×12×256	FC3	4096	7

**Table 2:** Data augmentation strategies and their parameter configurations.

Processing Method	FER2013	CK+
Rotation	±10°	±10°
Width Shift	±0.2	±0.05
Height Shift	±0.2	±0.05
Horizontal Flip	True	True
Shear Range	±0.2	±0.2
Zoom Range	±0.2	±0.2

**Table 3:** Accuracy Rates of different attention mechanisms on FER2013 and CK+ datasets.

Attention Mechanisms	FER2013 Accuracy (%)	CK + Accuracy (%)
ECANet	68.20	96.27
CANet	67.98	95.93
SENet	68.54	96.95
CBAM	71.52	97.97

**Table 4:** Comparison results of different facial expression recognition methods.

Dataset	Method	Accuracy (%)
FER2013	Improved YOLOv8 [40]	69.72
	CC-CNN [41]	71.16
	ShuffleNetV2 [42]	69.12
	CAGNet	71.52
CK+	CC-CNN [41]	97.73
	Gabor [43]	94.20
	Improved Residual Network [22]	96.37
	CAGNet	97.97

## Data Availability

The data supporting the findings of this study, including the CK+ and FER2013 datasets, are openly available at http://www.jeffcohn.net/Resources/ (for CK+, accessed on 15 May 2023) and https://www.kaggle.com/datasets/msambare/fer2013 (for FER2013, accessed on 17 August 2023).
